# Inter-device Agreement and Normative Corneal Parameters in Adolescents and Young Adults: A Structured Narrative Review

**DOI:** 10.7759/cureus.110307

**Published:** 2026-06-05

**Authors:** Stella Georgiadou, Costas H Karabatsas, Athina Plakitsi, Evangelos Pateras

**Affiliations:** 1 Optics and Optometry, Faculty of Health and Care Sciences, University of West Attica, Athens, GRC; 2 Optometry, LaserVision Ambulatory Eye Surgery Unit, Athens, GRC; 3 Ophthalmology, LaserVision Ambulatory Eye Surgery Unit, Athens, GRC; 4 Ophthalmology, Attiko Eye Center, Athens, GRC

**Keywords:** collegiate population, corneal imaging, corneal tomography, corneal topography, inter-device aggreement, normative corneal parameters, oct, placido, scheimpflug, school population

## Abstract

Accurate characterization of the corneal surface is essential for clinical decision-making in optometry and ophthalmology, particularly in the context of myopia management and early detection of ectatic disorders. Optical imaging technologies, including Placido-based topography, Scheimpflug tomography, and optical coherence tomography (OCT), are widely used to assess corneal geometry, yet their measurements are not fully interchangeable. This study was conducted as a structured narrative review that summarizes recent evidence (2020-2025) on inter-device agreement and synthesizes normative corneal geometric parameters in secondary school and university-aged populations.

A structured literature search identified studies evaluating agreement between two or more corneal imaging technologies, as well as studies reporting normative corneal parameters in individuals aged 12 to 25 years. Findings were synthesized narratively due to heterogeneity in study designs and reported outcomes. Thirty-five studies compared measurements across devices, and forty-five studies provided normative data. Most devices showed high correlation for central anterior keratometry (simulated keratometry (Sim-K); typically r>0.95), with consistent mean Sim-K differences of 0.10-0.25 D between Placido and Scheimpflug systems. Scheimpflug tomography generally reported steeper posterior curvature and greater posterior elevation than OCT, while OCT yielded corneal pachymetry values approximately 10-20 μm thicker than Scheimpflug tomography.

These systematic differences reflect the distinct measurement principles of each technology and underscore their non-interchangeability. Normative data from school-aged populations revealed meaningful variability in corneal curvature, asphericity, and thickness across ethnic and refractive groups, with East Asian cohorts typically exhibiting flatter corneas and less prolate asphericity. Overall, Scheimpflug and OCT technologies provide more comprehensive corneal assessment than Placido-based topography, particularly for posterior corneal geometry and pachymetry. Population-specific normative values and awareness of device-specific measurement differences are essential for accurate diagnosis, myopia management, and refractive surgery screening in adolescents and young adults.

## Introduction and background

As the main refractive surface of the eye, the cornea has a complex aspheric shape that is essential for normal visual function. Quantitative assessment of its surface topography and tomography, including parameters such as anterior and posterior curvatures, asphericity (Q-value), corneal thickness (pachymetry), and elevation maps, is a fundamental element of modern visual science [1]. The Q-value describes the rate at which corneal curvature flattens from the center toward the periphery (negative values indicate a prolate shape), while posterior elevation refers to the deviation of the posterior corneal surface relative to a best-fit sphere. 

These measurements have extensive clinical applications, including the diagnosis of keratoconus and other ectatic disorders, planning of refractive and cataract surgery, fitting of contact lenses (especially orthokeratology for myopia control), as well as monitoring of ocular manifestations of systemic diseases [2-12]. Over the past two decades, optical imaging technology for corneal evaluation has rapidly advanced. Placido-disc topographers assess the anterior tear film by analyzing reflected concentric rings, whereas rotating Scheimpflug cameras, e.g., Pentacam (Oculus Optikgeraete GmbH; Wetzlar, DEU), Galilei (Ziemer Ophthalmic Systems AG; Bern, CHE), and Sirius (Construzione Strumenti Oftalmici; Florence, ITA), acquire cross-sectional images to reconstruct a 3D model of the anterior segment. High-resolution optical coherence tomography (OCT) systems, e.g., Anterion (Heidelberg Engineering GmbH; Heidelberg, DEU), Cirrus HD-OCT (Zeiss; Oberkochen, DEU), and RTVue (Optovue Inc.; Fremont, CA, USA), use interferometry to generate detailed cross-sectional images with micron-level axial resolution [13-16]

The rapid expansion of technology raises an important question for clinicians and researchers: Can the geometric parameters obtained from different devices be applied interchangeably? Increasing evidence suggests that the answer is no, as systematic differences exist between these technologies [17-19]. This non-interchangeability is particularly significant for critical applications in specific demographic groups, such as secondary and university school-age populations (12 to 25 years). The 12 to 25 years age range was selected because it represents the period of active refractive development, rapid myopia progression [20,21], and represents a crucial window for interventions such as orthokeratology, early ectasia screening, and the use of collagen cross-linking in cases of established or suspected keratoconus. It also corresponds to the age at which many individuals undergo pre-refractive surgery evaluation, making accurate normative corneal data particularly relevant [22-24]. 

However, no recent review has synthesized both inter-device agreement and population-specific normative corneal geometry specifically in individuals aged 12 to 25 years, despite the increasing clinical relevance of this demographic. Accordingly, the aim of this structured narrative review is twofold: (1) to compare geometric parameters of the corneal surface obtained from optical imaging devices (Placido, Scheimpflug, and OCT), and (2) to present the distribution of key geometrical parameters in normative, school-aged populations, considering the variance due to age, ethnicity, and refractive status.

## Review

Methods

A structured literature search was conducted in PubMed, Scopus, Web of Science, and Embase to identify relevant publications from January 2020 to March 2025. The full search strategy included Boolean operators and combined the following corneal imaging terms (“corneal topography,” “corneal tomography,” “Scheimpflug,” “optical coherence tomography,” “keratometry”), population terms (“adolescents,” “young adults,” “students”), and filters restricting results to English language, human subjects, and individuals aged 12 to 25 years. An example PubMed search string was (“corneal tomography” OR “Scheimpflug” OR “corneal topography”) AND (“adolescents” OR “young adults”) AND (2020:2025[dp]). Duplicate records were automatically removed using database tools and manually verified during screening.

Studies were eligible for inclusion if they were original research involving human participants aged 12 to 25 years, used Placido-based topography, Scheimpflug tomography, or OCT, and reported either inter-device agreement or normative corneal geometric parameters. Studies were excluded if they did not report corneal geometric parameters; included participants outside the 12 to 25-year age range; were case reports, reviews, conference abstracts, or other forms of non-original research; used devices not based on Placido, Scheimpflug, or OCT imaging; or lacked sufficient methodological detail to extract relevant outcomes.

Screening Process

Two reviewers independently screened titles and abstracts, followed by full-text assessment. Disagreements were resolved by consensus. Reference lists of included studies were also screened to identify additional eligible publications.

Synthesis Approach

**Figure 1:**
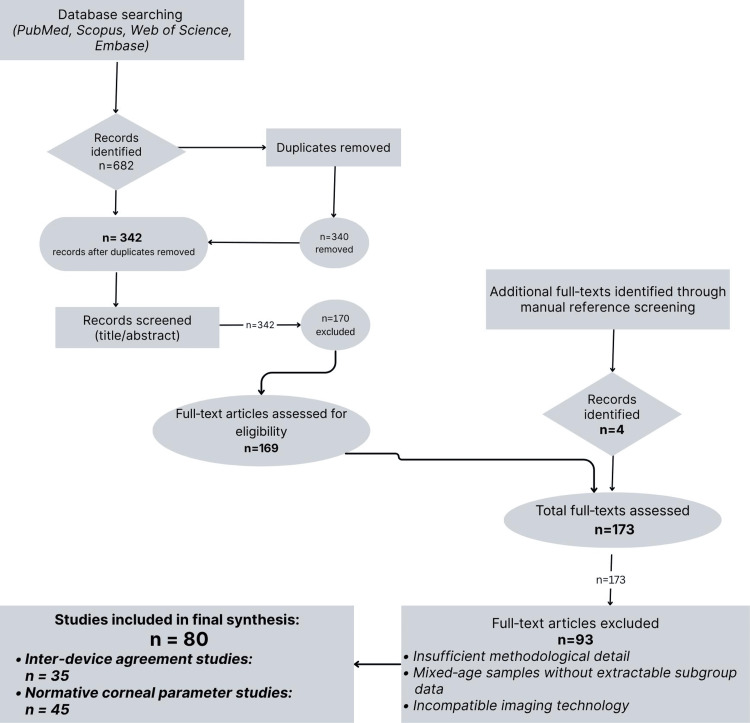
Study-selection flow diagram

Due to heterogeneity in study designs and outcomes, a meta-analysis was not feasible. A formal quantitative synthesis was not performed because the included studies differed substantially in device models, measurement protocols, reported parameters, and statistical outputs, preventing meaningful pooling of numerical data. Manual reference screening contributed to an additional four studies. A simplified study selection flow diagram summarizing the records identified, screened, excluded, and included is provided in Figure 1. Findings were synthesized narratively. No protocol was registered in the International Prospective Register of Systematic Reviews (PROSPERO) or a similar registry. Given the narrative nature of this review and the heterogeneity of the study designs, a formal risk of bias or study quality assessment (e.g., Quality Assessment of Diagnostic Accuracy Studies (QUADAS)-2) was not applicable and therefore not performed. 

Results

The literature search identified a large body of relevant publications, which were screened for eligibility. Ultimately, 80 studies satisfied the inclusion criteria and were incorporated into the final synthesis. Among these, 35 studies conducted direct comparisons between two or more corneal imaging technologies, while 45 studies provided data on normative corneal geometric parameters in populations aged 12 to 25 years. Due to heterogeneity in devices, acquisition protocols, and reporting formats, numerical values are presented as ranges across studies rather than pooled estimates. Consequently, the findings are divided into two sections: (I) inter-device agreement in corneal imaging and (II) the distribution of corneal geometric parameters in secondary school and university populations.

Inter-device Agreement in Corneal Imaging

Anterior corneal keratometry and astigmatism: With respect to standard keratometric values measured from the anterior central cornea (simulated keratometry (Sim-K): flat (K1), steep (K2), and mean (Km)), most of the 35 comparative studies reported a high correlation (r > 0.95) between Placido, Scheimpflug, and OCT measurements [25-30]. However, despite a high correlation, the Bland-Altman analyses consistently showed small but statistically significant mean differences [17,25,26].

Placido-based systems generally yielded slightly steeper central Sim-K values compared with Scheimpflug tomography, with reported mean differences ranging from 0.10 to 0.25 D. Biases greater than 0.25 D may be clinically relevant for refractive surgery planning [27,28]. Swept-source OCT devices, particularly Anterion, showed excellent agreement with Scheimpflug systems, with reported biases typically below 0.10 D, which are considered clinically insignificant for most routine applications [26,29]. Despite the high correlation, several studies reported wide limits of agreement, indicating that these devices are not fully interchangeable for keratometry [17,25,30].

Measurements of corneal astigmatism magnitude and axis also demonstrated good agreement across platforms, although it is well established that the disagreement in astigmatism axis between keratometry and Placido-based topography in low astigmatism (<1.5 D) is clinically significant and cannot be overlooked [29,31]. Furthermore, Scheimpflug and OCT devices have been reported to be more reliable in eyes with irregular astigmatism or unstable tear film conditions [9,25,26,32].

Corneal asphericity and peripheral curvature: The agreement between the devices was poorer for parameters describing the corneal shape beyond the central optical zone. Corneal asphericity (Q-value) showed substantial variability across technologies, with Scheimpflug systems generally reporting more negative Q-values (indicating a more prolate corneal shape) than Placido-based systems. These discrepancies are attributable to differences in the measurement principles, as Placido systems assess the anterior tear film surface, whereas Scheimpflug imaging evaluates the true corneal surface and stromal geometry. Another reason might be that Placido technology uses spherocylindrical algorithms to calculate Sim-K from the central 3 mm area [27,32,33].

Posterior corneal surface and pachymetry: The most clinically relevant inter-device differences were observed in the posterior corneal measurements and corneal thickness. Devices that do not measure the posterior surface of the cornea (i.e., Placido-based systems) do not directly image the posterior corneal surface and rely on theoretical assumptions, limiting their accuracy for posterior curvature assessment [34]. In contrast, Scheimpflug and OCT devices directly image the posterior cornea. Across comparative studies, Scheimpflug tomography tended to report steeper posterior curvatures and greater posterior elevations than OCT devices [26,29,35].

In addition, OCT-based systems generally reported central and thinnest corneal thickness (TCT) values that were generally 10-20 µm thicker than those obtained using Scheimpflug tomography [29,35,36]. These systematic differences reflect the distinct segmentation and boundary detection principles inherent to each technology and have important implications for ectasia screening, intraocular pressure (IOP) correction, and refractive surgery planning [35,37]. A consolidated overview of mean biases, limits of agreement, and correlation coefficients across device pairs is presented in Table 1.

**Table 1:** Summary of inter-device agreement across corneal imaging technologies Sim-K: Simulated keratometry; D: Diopters, OCT: Optical coherence tomography, IOP: Intraocular pressure

Device comparison	Mean bias	Correlation (r)	Clinical interpretation
Placido vs. Scheimpflug (Sim-K)	+0.10 to +0.25 diopters (D) (Placido steeper) [27,28]	>0.95	Not interchangeable for surgical planning; >0.25 D is clinically relevant
Scheimpflug vs. OCT (Sim-K)	<0.10 [26,29]	>0.95	Excellent agreement; differences clinically negligible
Scheimpflug vs. OCT (pachymetry)	OCT thicker by 10-20 μm [29,35,36]	High	Important for ectasia screening and IOP correction
Scheimpflug vs. OCT (posterior curvature)	Scheimpflug steeper [26,29,35]	Moderate-high	Device-specific normative databases required

Agreement in Specific Populations 

There was generally good agreement between the devices in school-aged myopes. However, in cases of high myopia and steeper corneal curvature, the limits of agreement broadened for peripheral measurements, indicating lower reliability in this population [27]. The choice of device significantly affects the lens selection parameters used for orthokeratology fitting, which predominantly relies on mid-peripheral corneal curvature measurements [4]. Regarding the distribution of geometrical parameters in school populations, 45 studies provided normative data on corneal geometry in populations aged 12 to 25 years, with the majority derived from cross-sectional school or university-based screenings. Across studies, mean keratometry ranged from 42.3 to 44.5 diopters (D), consistent with the variability observed between ethnic groups [38-41].

An association was consistently observed between flatter corneal curvature, greater myopia, and East Asian ancestry. Multiple studies from China, Singapore, and South Korea reported mean Km values of approximately 42.5 D to 43.0 D [42-44]. Middle Eastern and European populations showed slightly steeper mean values, ranging from 43.5 D to 43.8 D [38,39,45].

Prevalence of corneal astigmatism (≥ 0.75 D), ranging from approximately 40% to 60% [46], with against-the-rule astigmatism increased with age. Across studies, with-the-rule astigmatism predominated in early adolescence, while a gradual shift toward against-the-rule astigmatism was consistently observed in late adolescence and early adulthood [38,39,47,48].

Mean corneal asphericity (Q-value) across all studies was approximately −0.23, with a considerable standard deviation, indicating substantial biological variability. Corneal asphericity is associated with corneal curvature, as flatter corneas, often correlated with axial myopia, tend to be less prolate (Q-values closer to zero) [33,40]. Ethnic differences have also been reported, with some African and Middle Eastern populations exhibiting more prolate corneal profiles than Asian populations [42,45]. These findings are relevant for evaluating higher-order aberrations and overall optical quality in young populations [41].

The mean TCT was found to range between 505 and 555 µm. Thicker corneas were weakly associated with male sex and darker iris colors [38,43,49]. Multiple large studies have reported little to no correlation between axial length (and therefore degree of myopia) and central corneal thickness, suggesting that structural changes associated with myopia predominantly affect the sclera rather than the cornea [50-53].

The mean posterior radius of curvature ranged from 6.2 mm to 6.4 mm, whereas in Scheimpflug-based studies, the posterior elevation at the thinnest point of the cornea typically ranged from +5 µm to +15 µm relative to the best-fit sphere. These parameters are increasingly used as baseline reference values for detecting subclinical ectasia during the preoperative screening of university-aged candidates for refractive surgery [54,55]. Screen time was associated with dry eye and transient irregularities affecting measurement repeatability [56-58]

Because of heterogeneity in reporting formats across studies, pooled confidence intervals could not be calculated; therefore, ranges are presented to reflect the variability of findings. Reported ranges for normative values across studies are summarized in Table 2.

**Table 2:** Reported ranges of normative corneal parameters in individuals aged 12 to 25 years Sim-Km: Simulated keratometry mean, D: Diopters; TCT: Thinnest corneal thickness; OCT: Optical coherence tomography

Parameter	Range	Notes
Sim-Km (D)	42.3-44.5 D [38-41]	Flatter in East Asian cohorts (42.5-43.0 D) [42-44][; slightly steeper in Middle Eastern/European populations (43.5-43.8 D) [38,39,45]
Astigmatism ≥ 0.75 D (%)	40%-60% [46]	Against-the-rule astigmatism increases with age [38,39,47,48]
Qvalue (asphericity)	Mean ≈ −0.23 (substantial variability across studies)	Flatter corneas (often myopic) show less prolate profiles (Q closer to 0) [33,40], values vary significantly across ethnic groups and refractive profiles [42,45]
TCT	505-555 μm	Slightly thicker in males and darker irides [38,43,49]
Posterior radius of curvature	6.2-6.4 mm	Derived mainly from Scheimpflug/OCT studies [54,55]
Posterior elevation (μm)	+5 to +15 μm	Scheimpflug-based studies measured at the thinnest point relative to the best-fit sphere [54,55]

Discussion

This structured narrative review summarizes recent evidence on corneal geometric parameters and inter-device agreement in adolescents and young adults. Contemporary corneal imaging devices generally show high correlation for anterior keratometry and anterior chamber parameters. However, small but consistent inter-device differences were reported across studies, and these should be interpreted within device-specific normative frameworks rather than assumed to represent fixed measurement biases.

Placido-disc topographers provide excellent assessment of the anterior corneal surface; however, they do not generate tomographic data and, as well documented in the literature, are insufficient for comprehensive ectasia risk screening [6,7]. In contrast, Scheimpflug tomography and swept-source OCT tend to enable a more complete corneal evaluation by directly imaging the posterior corneal surface and generally providing more consistent pachymetric measurements. Although correlation between devices was often high, limits of agreement were frequently wide, indicating that several instruments cannot be used interchangeably for keratometry or pachymetry [17,25,30]. The consistent measurement bias observed between the Scheimpflug and OCT systems for posterior curvature and corneal thickness highlights the importance of device-specific normative databases. Accordingly, longitudinal patient follow-up and the development of surgical nomograms should be based on measurements obtained using the same imaging platform [59,60].

Clinical Implications

Beyond inter-device agreement, an important contribution of this review lies in the synthesis of normative corneal geometric data derived from school-aged and university-level populations. Normative data demonstrated substantial variability in corneal geometry across different ethnicities and refractive profiles. The flatter corneal curvature and distinct asphericity patterns observed in East Asian myopic populations are not merely epidemiological findings but have direct clinical relevance for optical design, particularly in applications such as myopia-control spectacles and orthokeratology lens fitting. Similarly, the establishment of ethnicity-specific normative ranges for robust tomographic parameters, including posterior corneal elevation, represents an important step toward improving the early detection of subclinical ectasia and reducing the risk of post-LASIK ectasia in young adults, an increasingly represented group within the refractive surgery population [4,5,54,55,61-66]. Device-specific normative databases remain essential for accurate interpretation, and longitudinal follow-up should ideally be performed with the same imaging platform. Central anterior keratometry showed the highest inter-device agreement, whereas posterior corneal parameters and pachymetry demonstrated lower interchangeability. These distinctions are important when interpreting measurements across different platforms. Environmental factors such as screen time may transiently affect tear film stability and measurement repeatability in adolescents and young adults [56,58].

Limitations

This review was conducted as a structured narrative synthesis rather than a formal systematic review. As such, the full PRISMA methodology was not applied because the substantial heterogeneity in study designs, measurement protocols, and reported outcomes made standardized extraction and meta-analytic pooling inappropriate. Consequently, values are presented as reported ranges rather than pooled estimates. This methodological choice may introduce selection bias, and although a structured search strategy was used, the absence of PRISMA flow elements limits the full reproducibility. Some of the included studies had relatively small sample sizes, limiting the ability to perform detailed subgroup analyses. Additionally, heterogeneity in study designs, measurement protocols, and reporting formats prevented the use of meta-analytic techniques. Differences in population characteristics and device-specific normative databases may also influence the generalizability of the findings. Another challenge is the rapid evolution of corneal imaging technology. Frequent software updates and new device models may influence measurement consistency over time and complicate longitudinal comparisons, which inherently carry a risk of selection bias. No formal risk-of-bias assessment was performed, and substantial heterogeneity in devices, acquisition protocols, and reporting formats limited direct comparability across studies.

Future Directions

Future research would benefit from large-scale, multi-ethnic, longitudinal studies that track children and adolescents throughout their school years, integrating detailed corneal geometric evaluations with simultaneous measurements of axial elongation. Such study designs could help identify whether specific corneal topographic or tomographic patterns predict rapid myopia progression [67,68]. Understanding device-specific measurement differences is essential when monitoring young patients undergoing myopia management or refractive surgery evaluation. Consistent use of the same imaging platform is recommended for longitudinal follow-up, as cross-device comparisons may introduce systematic biases in the measurements.

Further efforts are required to improve the standardization of corneal imaging platforms. Emerging approaches, including artificial intelligence-based algorithms, may facilitate the translation of measurements between devices or enable the development of device-independent risk scores [69]. Integrating corneal geometric parameters with axial length, lens thickness, and additional ocular metrics within a comprehensive 'whole-eye' model could further enhance the understanding of refractive development and disease risk in young populations [70-74].

## Conclusions

The evaluation of corneal surface geometry has been transformed by the development of various optical imaging technologies. This review suggests that Scheimpflug and OCT instruments provide a more comprehensive and dependable assessment than Placido-based systems, particularly for the posterior cornea and pachymetry, with important and clinically meaningful differences existing even between these types of advanced technologies. In secondary school and university-level populations, normative corneal parameters showed substantial variability attributable to ethnicity and refractive error, underscoring the need for population-specific reference values. For clinicians and public health practitioners, device-specific knowledge and normative data are essential for accurate diagnosis, appropriate myopia management, and safe refractive surgery screening in adolescents and young adults. Clinicians are advised to interpret cross-device comparisons with caution during longitudinal follow-up and interpret measurements within the context of known device-specific biases.
